# Supplementary figures and images for: Analysis of the resistance level and target site resistance mechanisms of Echinochloa crus-galli to penoxsulam from Hubei Province, China
Source: PeerJ. 2025 Sep 8;13:e19973. doi: 10.7717/peerj.19973 (PMC12424613; doi:10.7717/peerj.19973)

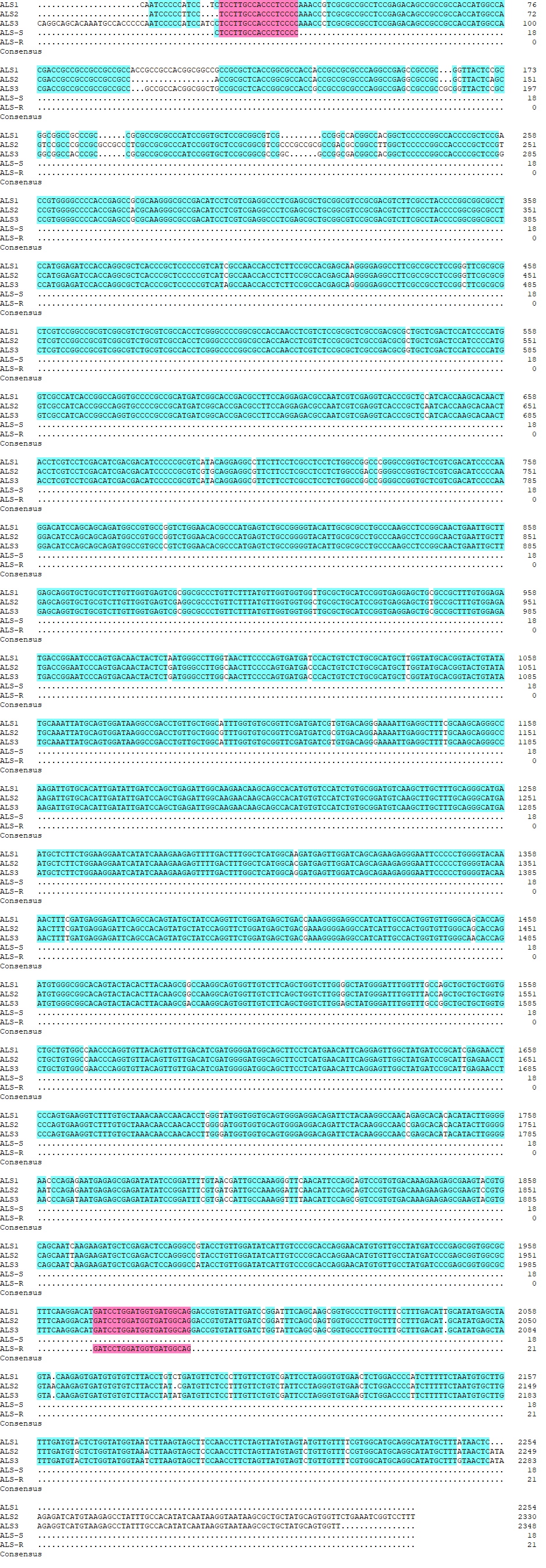

Supplement: Supplemental Information 5 [file peerj-13-19973-s005.jpeg]
